# Evaluation and lessons learned from the dissemination and implementation science scholars program in the national cancer prevention and control research network

**DOI:** 10.1017/cts.2024.625

**Published:** 2024-10-29

**Authors:** Daniela B. Friedman, Cam Escoffery, Elaine H. Morrato, Cynthia A. Thomson, Courtney N. Petagna, Freda Allyson Hucek, Mary Wangen, Aubrey Villalobos, James R. Hebert, Samuel Noblet, Mayank Sakhuja, David O. Garcia, Jennifer L. Cruz, Stephanie B. Wheeler

**Affiliations:** 1 Department of Health Promotion, Education, and Behavior, Arnold School of Public Health, University of South Carolina, Columbia, SC, USA; 2 Department of Behavioral, Social, and Health Education Sciences, Rollins School of Public Health, Emory University, Atlanta, GA, USA; 3 Parkinson School of Health Sciences and Public Health, Loyola University Chicago, Chicago, IL, USA; 4 Department of Health Promotion Sciences, Mel & Enid Zuckerman College of Public Health, University of Arizona, Tucson, AZ, USA; 5 Center for Health Promotion and Disease Prevention, University of North Carolina at Chapel Hill, Chapel Hill, NC, USA; 6 Division of Cancer Control and Population Sciences, National Cancer Institute, National Institutes of Health, Rockville, MD, USA; 7 Department of Epidemiology and Biostatistics & Cancer Prevention and Control Program, Arnold School of Public Health, University of South Carolina, Columbia, SC, USA; 8 Envera Health, Richmond, VA, USA; 9 UNC Lineberger Comprehensive Cancer Center, University of North Carolina at Chapel Hill, Chapel Hill, NC, USA; 10 Department of Social and Behavioral Sciences, Harvard T.H. Chan School of Public Health, Boston, MA, USA; 11 Department of Health Policy and Management, Gillings School of Global Public Health, University of North Carolina at Chapel Hill, Chapel Hill, NC, USA

**Keywords:** Capacity building, co-development, research training, cancer prevention and control, implementation science

## Abstract

**Background:**

The Centers for Disease Control and Prevention (CDC)-funded Cancer Prevention and Control Research Network (CPCRN) has been a leader in cancer-related dissemination & implementation (D&I) science. Given increased demand for D&I research, the CPCRN Scholars Program launched in 2021 to expand the number of practitioners, researchers, and trainees proficient in cancer D&I science methods.

**Methods:**

The evaluation was informed by a logic model and data collected through electronic surveys. Through an application process (baseline survey), we assessed scholars’ competencies in D&I science domains/subdomains, collected demographic data, and asked scholars to share proposed project ideas. We distributed an exit survey one month after program completion to assess scholars’ experience and engagement with the program and changes in D&I competencies. A follow-up survey was administered to alumni nine months post-program to measure their continued network engagement, accomplishments, and skills.

**Results:**

Three cohorts completed the program, consisting of 20, 17, and 25 scholars in Years 1-3, respectively. There was a significant increase in the total D&I competency scores for all three cohorts for 4 overarching domains and 43 subdomains (M_Pre_ = 1.38 M_Post_ = 1.89). Differences were greatest for the domain of Practice-Based Considerations (0.50 mean difference) and Theory & Analysis (0.47 mean difference). Alumni surveys revealed that scholars appreciated access to D&I-focused webinars, toolkits, and training resources. 80% remain engaged with CPCRN workgroups and investigators.

**Conclusions:**

Program evaluation with scholars and alumni helped with ongoing quality assurance, introspection, and iterative program adaptation to meet scholars’ needs. This approach is recommended for large-scale capacity-building training programs.

## Introduction

### Background

#### Need for dissemination and implementation science training in cancer prevention and control

Dissemination and implementation (D&I) science entails studying how evidence-based practices, interventions, and policies can be translated into real-world settings to improve public health outcomes [1]. Cancer prevention and control training programs with a D&I focus have been developed by federal agencies and through federally funded grant programs [2,3] to increase our nation’s D&I research capacity to translate health evidence more rapidly into practice. There has been D&I programming for practitioners and public health scientists [1,4–8]. A major motivation for the National Cancer Institute’s (NCI) interest in D&I research is the fact that, while tens of billions of dollars have been spent on basic research, epidemiologic, and clinical research combined, many potentially relevant findings have not been translated into clinical or public health action [9–11]. A well-trained workforce is an important limiting factor in disseminating and implementing scientifically valid research findings [12]. Therefore, intentional efforts to increase training to address D&I needs while also focusing on growing a diverse workforce in D&I science is critical for improving cancer outcomes and reducing cancer-related health disparities [13,14].

The Centers for Disease Control and Prevention (CDC)-funded Cancer Prevention and Control Research Network (CPCRN) has emerged as a leader in cancer-related D&I science [15,16]. While the CPCRN has been in existence since 2002 (2002–2024), it is only in the past decade that the network has focused heavily on D&I science [16]. This has coincided with a deliberate and decisive move into community-based participatory research (CBPR) [16–18]. CBPR complements D&I science because effective D&I requires community involvement and support, especially if programs are intended to be sustained over long periods of time [19–21].

Given increased demand for D&I research, the CPCRN Scholars Program was developed in 2020 and launched in 2021 to expand the number of practitioners, researchers (including early-stage investigators), and students (e.g., graduate students and postdoctoral fellows) trained in cancer D&I methods [22]. Emphasis in training and education was placed on D&I methods as a way to address and prioritize cancer health equity research and practice [23]. In addition, the program uses both a tailored and co-learning approach for researchers and practitioners [24]. It also emphasizes translating research into practice through collaborative partnerships and the application of CBPR principles and partner engagement [9,22,25].

#### Overview of the CPCRN and scholars program

The CPCRN is comprised of eight funded academic centers across the United States. Network members include researchers, public health professionals, and federal and community partners. The members and affiliate members of the network apply evidence-based interventions and strategies to reduce the burden of cancer, especially those disproportionately affected [15]. To effectively do this, the CPCRN also focuses on developing a workforce of a broad array of professions in cancer prevention and control focused on D&I science as part of its mission. Recognizing the need to grow the pool of student, researcher, and practitioner scholars focused on D&I science, the CPCRN created an annual scholars training program beginning in 2020.

#### Program development

The CPCRN Scholars Program was developed using a collaborative workgroup approach. The Scholars Program Planning Workgroup was coordinated by University of North Carolina at Chapel Hill (UNC) Coordinating Center staff and lead investigators at Emory University and the University of South Carolina with collaborators at the University of Arizona, Colorado School of Public Health, Loyola University, and federal agency partners at the CDC and NCI. The group also engaged representative investigators from across the network sites to initiate the program development process and assist in the evaluation design. A logic model was used to guide the implementation and evaluation of all components of this training program [22].

#### Instructional design

Given that this is a national network with funded centers across eight states and affiliate members with widespread geographical representation, meetings, and trainings have occurred mostly via Zoom (other than annual in-person meetings). Program and curriculum development was guided by a prior CDC-funded student-focused scholars program on brain health [26,27], an in-depth formative evaluation, and capacity-building expertise of network members [3,22,25].

The program curriculum, consisting of readings and videos that were posted on a virtual platform (Trello) for each scholar, focused on evidence-based public health/cancer interventions and D&I scientific frameworks, strategies, and methods to advance knowledge and increase competencies related to implementation research and practice. As part of this, within a 12-month period (this was condensed to 9 months for the first program cohort), scholars had to complete one of two D&I curricula and the curricula chosen were based on what track the scholar chose to be in at the beginning of the program. The curricula were: (1) the *Putting Public Health Evidence into Action Training* developed by CPCRN investigators [3,28] and (2) the NCI’s *Training Institute for Dissemination and Implementation Research in Cancer* (TIDIRC), open-access modules [2]. In addition, scholars were asked to develop or participate in a project that provided experiential learning in terms of D&I principles and practices.. Often these were cross-center CPCRN projects based within workgroups; scholars could also conduct projects at their local CPCRN site, and some student scholars considered their thesis/dissertation to be the CPCRN project requirement. The mentors the scholars identified at the beginning of the program assisted their scholar throughout the scholar’s project. Scholars typically had 1–2 mentors and a mentor may have had multiple scholars. The projects could have involved other scholars or CPCRN investigators, particularly if the project was developed in a CPCRN workgroup. The scholars presented their projects to the Scholars Planning Workgroup, Scholar Program leadership team, and the Coordinating Center during end-program activities.

Scholars across all tracks had the opportunity to interact and learn from each other during scheduled virtual discussions and meet-ups. Together, they also attended virtual webinars on topics such as D&I theories and frameworks, implementation in action, implementation in a global context, scoping and systematic reviews, and D&I in real-world settings. Scholars participated in local and CPCRN network-wide meetings, and meetings with mentors and other scholars [22,25]. We had a kickoff and closing meeting for each cohort. Following the program, scholars received certificates of completion.

#### Scholar recruitment and selection

The Scholars Program Planning Workgroup drafted an email calling for applications, which was sent to members of the CPCRN Steering Committee consisting of principal investigators, project directors, and workgroup leads. Additionally, the application was sent to the overall Network listserv with a request to distribute the email and link to an electronic application form. Network members distributed the email via student and researcher listservs at their home institutions; affiliates and federal agency partners (CDC and NCI) also shared the information. Individual investigators and the CPCRN Coordinating Center (UNC-Chapel Hill) shared the opportunity on social media. As the program progressed with new cohorts in each of the second and third years of the program, alumni of the program shared the call with their networks and assisted with new scholar recruitment and mentorship.

Two Scholar Program Planning Workgroup reviewers were assigned to each application received. Reviewers were selected based on their familiarity with the CPCRN structure and content expertise. This made it feasible to link scholars with interests with expertise residing across the CPCRN and within specific workgroups (e.g., Survivorship, Health Behaviors). A rubric was used to rate applications on individuals’ evidence of interest in cancer prevention and control and/or D&I science; a clear, concise description of a proposed project; a clear description of the proposal goals and activities; feasibility of completing the proposed project in the one-year timeframe; how proposed goals and activities contributed to the diversity of the training program; and how the proposed goals and project aligned with CPCRN efforts. Applicants were asked to recommend program mentors who were typically at their local CPCRN site or affiliate institution and/or workgroup members. However, this was not a strict requirement as some applicants were completely unaffiliated with any CPCRN center. Additional details about program development, recruitment, and review of applications can be found elsewhere [22,25].

#### Study objective

The objective of this paper is to summarize lessons learned from the first three training cohorts (*N* = 63 scholars) of the CPCRN Dissemination and Implementation Science Scholars Program (2020–2023). Detailed information about the early development of the CPCRN Scholars Program, including structures and processes, can be found in previously published articles [22,25]. In the following sections, we describe the program evaluation data and lessons learned for other research networks implementing similar scholars programs.

## Materials and methods

### Program evaluation

For the three cohorts, the scholars program application also served as the baseline survey, and it included demographic questions, names of current mentors, CPCRN workgroups of interest, a proposed project description, one to three professional development goals, related activities they had for participation, and how their proposed work aligned with the CPCRN Strategic Plan and/or Logic Model.

Additionally, applicants were asked to self-rate their competencies in D&I science (1 = beginner, 2 = intermediate, 3 = advanced) based on published descriptions of competencies and similar Likert scoring [29]. Specifically, scholars self-reported their competencies focused on D&I background knowledge (10 subdomain items), theory and approaches (7 items), study design and analysis (14 items), and practice-based considerations (12 items). The comprehensive list of competencies was developed using a multi-phase approach that included expert consensus identification of individual competencies followed by card sorting, an acknowledged, effective approach to categorize knowledge [30], organize domains, and establish subdomain items for inclusion under each domain [31]. These competencies have been implemented widely in the development of the Mentored Training for Dissemination and Implementation Research in Cancer (MT-DIRC) Program at Washington University in St Louis and have been used in several other D&I-focused training programs [32,33]. These self-rated competencies specifically include relevant practice-based competencies which are especially important for our CPCRN Scholars program given the critical involvement and our focus on recruitment of practitioners (in addition to academics) and the strong interest of students and faculty members in the applied, real-life implementation of evidence-based strategies and programs.

Following the completion of the program, scholars received a post-program survey that included self-rated competency questions similar to the pre-program assessment. Response rates varied for this survey by cohort, ranging from 88% (cohort 2) to 92% (cohort 3). After the first cohort completed the program, we also conducted interviews with scholars to understand more in-depth their satisfaction with and recommendations for the program. Findings from these qualitative interviews of the first cohort can be found elsewhere [25]. In addition, an alumni survey was distributed to scholars (from cohorts 1–2 thus far) nine months post-program. It consisted of 13 questions, of which five items allowed for open-ended responses specifically regarding their projects, continued network engagement, accomplishments, and skills. The alumni survey asked about current employer information/position title, graduation information (if the scholar was a student while pursuing the training program), current level of engagement with CPCRN workgroups and interest in remaining engaged with CPCRN in a scholar alumni role, peer-reviewed publications, conference presentations, and/or grants submitted or funded since their time in the program, type of continued engagement with D&I science and/or cancer prevention and control, interest in serving as a mentor for a future CPCRN scholar, and skills they learned and/or wished they learned from the program. Descriptive statistics (frequencies, percentages) and paired t-tests were calculated using SPSS v27.

## Results

### Program participation

**Figure 1.**
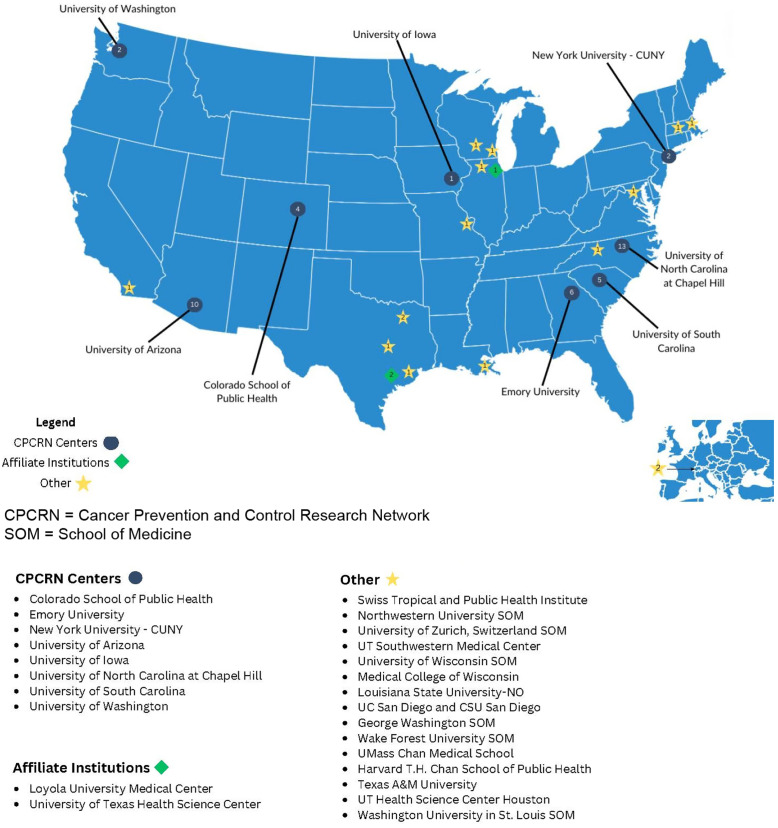
Distribution of scholars across CPCRN centers and other institutions.

To date, the program has been implemented with three cohorts. Year 1 had 20 scholars (24 applicants) and 30 mentors; Year 2 had 17 scholars (19 applicants) and 15 mentors, and Year 3 had 25 (2 had to drop the program; 47 applicants) and 38 mentors. Year 3 demonstrated a 135% increase in applications from the previous year. Scholars came from 21 schools and organizations within the United States and abroad (Switzerland). Scholars were diverse and had good representation across our categories of audiences (e.g., students, practitioners). Scholars’ disciplines have mainly been in public health (including health promotion, epidemiology, health policy, and management), health sciences, and behavioral and social sciences. Table 1 presents detailed demographics about participating scholars; Table 2 presents scholars by track for each year of the program; and Figure 1 presents the number of scholars by year, geographic location, and institution (CPCRN center or affiliate center, or other). The final year of the program for this specific CPCRN grant cycle (2019–2024) involved scholar alumni from all years of the program (See Lessons Learned section for additional details).

**Table 1.** Scholar demographics and characteristics (N = 62)

Variable	Pre-program survey *N* (%)
Cohort	
1	20 (32.3%)
2	17 (27.4%)
3	25 (40.3%)
Scholar type	
Researcher/Practitioner	34 (54.8%)
Student	28 (45.2%)
*Student (of n = 28)*	
In what type of degree-granting program are you currently enrolled?	
PhD	24 (85.7%)
MPH	2 (7.1%)
MSPH	1 (3.6%)
MPH/MSW	1 (3.6%)
What year of the program are you in?	
First	8 (28.5%)
Second	2 (7.1%)
Third	10 (35.7%)
Fourth	5 (17.8%)
Final	1 (3.5%)
Gender	
Female	50 (80.6%)
Male	12 (19.4%)
Race	
White	34 (54.8%)
Asian	19 (30.6%)
Black or African American	8 (12.9%)
Mexican American	1 (1.6%)

**Table 2.** Number of scholar program participants by career track

Cohort	Student/Postdoctoral	Faculty/Researcher	Practitioner	Total Scholars
1 (2021)	10	9	1	20
2 (2022)	7	3	7	17
3 (2023)	11	14	0	25
**Total**	**28**	**26**	**8**	**62**

### Overall program evaluation and impact

We present baseline and post-program survey data from all three scholar cohorts, and alumni evaluation data from two cohorts, including qualitative data captured at the end of the first yearof the Scholars Program. Scholars to date (*N* = 62) were mostly students (45.2%) or researchers (42.0%) across the three cohorts (Tables 1 and 2). They were mostly female (80.6%) and racially diverse (45.2%).

Table 3 presents the merged baseline and post-program survey data combined for the three Scholars Program cohorts. Scholars rated greater overall experience with D&I science after the program (M_Pre_ = 1.38, M_Post_ = 1.89). There were significant increases in the total D&I competency score for 100% of the 4 overarching domains (M_Pre_ = 1.49, M_Post_ = 1.99) and 43 subdomains. For each of the specific competency categories (A. Definition, Background, and Rationale, B. Theory and Approaches, C. Design & Analysis, and D. Practice-Based Considerations), scholars reported significant improvements from baseline to post-program. These differences were greatest for the domain of Practice-Based Considerations (0.50 mean difference) followed by Theory & Analysis (0.47 mean difference).

**Table 3.** Pre-/Post-scholar self-ratings on D&I science competencies*

Variable (Rated as Beginner, Intermediate, Advanced)	Pre-program survey Mean (*n* = 62)**	Post-program survey Mean (*n* = 57)	Mean difference
**Overall level of experience with dissemination and implementation science?**	1.38	1.89*	0.51
**Section A: Definition, Background, and Rationale**			
A1. Define and communicate D&I research terminology	1.69	2.08*	0.39
A2. Define what is and what is not D&I research	1.75	2.12*	0.37
A3. Differentiate between D&I research and other related areas, such as efficacy research and effectiveness research	1.62	2.12*	0.50
A4. Identify the potential impact of disseminating, implementing, and sustaining effective interventions	1.73	2.06*	0.33
A5. Describe the range of expertise needed to conduct D&I research	1.50	1.90*	0.40
A6. Determine which evidence-based interventions are worth disseminating	1.62	2.04*	0.42
A7. Assess, describe, and quantify (where possible) the context for effective D&I	1.42	1.92*	0.50
A8. Identify existing gaps in D&I research	1.38	1.85*	0.47
A9. Identify the potential impact of scaling down (aka de-implementing) an ineffective but often used intervention	1.19	1.79*	0.60
A10. Formulate methods to address barriers of D&I research	1.33	1.88*	0.55
**Overall Competency A**	**1.52**	1.97*	**0.45**
**Section B: Theory and Approaches**			
B1. Describe a range of D&I strategies, models, and frameworks	1.62	1.96*	0.34
B2. Identify appropriate conceptual models, frameworks, or program logic for D&I change	1.56	1.92*	0.36
B3. Identify core elements (effective ingredients) of effective interventions, and recognize risks of making modifications to these	1.42	1.90*	0.48
B4. Describe a process for designing for dissemination (planning for adoption, implementation, and sustainability during the intervention development stage)	1.40	1.98*	0.58
B5. Describe the relationships between various organizational dimensions (e.g., climate, culture) and D&I research	1.29	1.90*	0.61
B6. Explain how knowledge from disciplines outside of health (e.g., business, marketing, and engineering) can help inform further transdisciplinary efforts in D&I research	1.33	1.79*	0.46
B7. Identify and articulate the interplay between policy and organizational processes in D&I	1.27	1.77*	0.50
**Overall Competency B**	**1.41**	1.88*	**0.47**
**Section C: Design & Analysis**			
C1. Describe the core components of external validity and their relevance to D&I research	1.44	2.02*	0.58
C2. Identify common D&I measures and analytic strategies relevant for your research question(s)	1.42	1.96*	0.54
C3. Identify and measure outcomes that matter to stakeholders, adopters, and implementers	1.60	2.04*	0.44
C4. Describe the application and integration of mixed-method (quantitative and qualitative) approaches in D&I research	1.63	2.13*	0.50
C5. Apply common D&I measures and analytic strategies relevant for your research question(s) within your model/framework	1.33	1.87*	0.54
C6. Identify possible methods to address external validity in study design reporting and implementation	1.37	1.79*	0.42
C7. List the potential roles of mediators and moderators in a D&I study	1.37	1.83*	0.46
C8. Identify and articulate the tradeoffs between a variety of different study designs for D&I research	1.33	1.77*	0.44
C9. Describe how to frame and analyze the context of D&I as a complex system with interacting parts	1.19	1.81*	0.62
C10. Effectively integrate the concepts of sustainability/sustainment and the rationale behind them in D&I study design	1.29	1.65*	0.36
C11. Describe gaps in D&I measurement and critically evaluate how to fill them	1.27	1.71*	0.44
C12. Effectively explain and incorporate concepts of de-adoption and de-implementation into D&I study design	1.12	1.58*	0.46
C13. Incorporate methods of economic evaluation (e.g., implementation costs, cost-effectiveness) in D&I study design	1.21	1.52*	0.31
C14. Evaluate and refine innovative scale-up and spread methods (e.g., technical assistance, interactive systems, novel incentives, and “pull” strategies)	1.10	1.50*	0.40
**Overall Competency C**	**1.33**	1.79*	**0.46**
**Section D: Practice-Based Considerations**			
D1. Describe the importance of incorporating the perspectives of different stakeholder groups	2.15	2.44*	0.29
D2. Describe the concept and measurement of fidelity	1.62	2.13*	0.51
D3. Articulate the strengths and weaknesses of participatory research in D&I research	1.62	2.10*	0.48
D4. Determine when engagement in participatory research is appropriate with D&I research	1.42	2.08*	0.66
D5. Describe the appropriate process for eliciting input from community-based practitioners for adapting an intervention	1.69	2.02*	0.33
D6. Identify and apply techniques for stakeholder analysis and engagement when implementing evidence-based practices	1.40	1.90*	0.50
D7. Identify a process for adapting an intervention and how the process is relevant to D&I research	1.37	1.92*	0.55
D8. Explain how to maintain fidelity of original interventions during the adaption process	1.27	1.92*	0.65
D9. Identify sites to participate in D&I studies, and negotiate or provide incentives to secure their involvement	1.40	1.85*	0.45
D10. Identify and develop sustainable partnerships for D&I research	1.46	1.92*	0.46
D11. Describe how to measure successful partnerships for D&I research	1.23	1.79*	0.56
D12. Use evidence to evaluate and adapt D&I strategies for specific populations, settings, contexts, resources, and/or capacities	1.31	1.90*	0.59
**Overall Competency D**	**1.49**	1.99*	**0.50**

D&I = dissemination and implementation.

* *p < 0.05 for paired samples means test.*

** *Scale ranged from 1-3 with 1=beginner, 2=intermediate, 3=advanced.*

### Scholar alumni data and reflections

Fifteen of the 37 scholars responded (40.5% response rate). From the surveys, over half of the respondents (*n* = 8, 53.3%) were in new positions since completing the scholars program; 20.0% (*n* = 3) had graduated from a degree program since participating in the program. Eighty percent remained engaged with a CPCRN workgroup and/or still collaborated with a CPCRN investigator(s) and 86.7% (*n* = 13) had completed their program projects. Forty percent of scholar alumni (*n* = 6) expressed interest in serving as a mentor for a future CPCRN scholar and 33.3% (*n* = 5) offered to help plan upcoming events and initiatives. From the alumni survey, one alumna of the program served as a mentor to other scholars in future years of the program; another alumnus is now serving as a multiple principal investigator of a CPCRN Collaborating Center; and a third alumnus proposed a new interest group focused on LGBTQ + Health as part of the Health Equity workgroup and has taken on a leadership role with the network.

When asked what new skills they received from the program, most alumni mentioned the benefits of learning about theoretical and conceptual frameworks, and they appreciated the access to D&I-focused webinars, toolkits, and training resources. They also benefited from networking with other professionals and students and collaborating with them on academic products. When asked what they wished they had received or done in the program, we received five comments from alumni requesting content about sustainability planning (a webinar is being planned for our all-cohort professional development year), jobs outside of the academic space, and cancer-specific funding mechanisms. One respondent wished they had been more active in reaching out to their mentors outside of their institution and hoped there would be more collaborations across workgroups.

The main themes that emerged from the open-ended questions on the alumni survey related to scholars reporting being able to apply theoretical and conceptual frameworks to their D&I initiatives. They also gained knowledge and skills regarding the implementation and evaluation of evidence-based programs in their cancer prevention and control work and for their dissertation or projects, if they were students. Table 4 presents representative quotes from scholars on how they continued to use the content and/or skills they learned in the program.

**Table 4.** Scholar alumni reflections on how they are using content and skills from the program

*Both directly and indirectly the skills garnered during the CPCRN Scholars Program and after (now as an Alumni and still part of CPCRN) have afforded continuing learning opportunities for me in terms of gaining knowledge of the application of evidence-based approaches to community-based cancer prevention and health equity research. Indirectly the interest of remaining informed was implanted during my time as a Scholar, and thus, I now keep up with the literature of those in the network and beyond in an effort to continue learning. Directly, the opportunity to continue participating in workgroups has provided collaborative opportunities that also furthers knowledge and skills in the areas of cancer prevention and health disparities and health equity research.* – Faculty Researcher
*I’m continuing to focus on the implementation of evidence-based programs to support cancer screening in lower-resourced settings. This includes the selection and use of implementation strategies and theories, models, and frameworks to guide implementation and sustainment.* – Postdoctoral Fellow
*Applying information learned from TIDIRC modules to personal grant proposals. Leveraging information from PPHEA modules and webinars to build and sustain relationships with school and community partners.* – Postdoctoral Fellow
*My work is focused on supporting safety net clinic systems (FQHCs and rural health clinics) by implementing and evaluating evidence-based interventions and approaches for colorectal cancer screening, as well as lung cancer screening and hereditary cancer risk assessment. Therefore, the CPCRN Scholars curriculum provided formal, comprehensive training in D&I to support this work.* – Practitioner (Program Manager)
*I am using lots of information learned related to implementation science and dissemination in my dissertation and an implementation science grant I am currently working on.* – PhD Student
*The CPCRN Scholars Program is an incredible program and I’m thankful for the opportunity to be a CPCRN Scholar. My knowledge on implementation science and cancer health equity has broadened and I’m excited to bring these concepts into my research. I will also cherish connecting with folks from the Scholars Program and the CPCRN network throughout the year at various events.* – Postdoctoral Fellow
*As a CPCRN Scholar, I not only strengthened my skill sets in D&I science but also learned about the importance of developing and maintaining multidisciplinary collaborations in order to improve cancer prevention and control. The opportunity to learn from faculty and peers outside of my field was especially invaluable, and I look forward to engaging in cancer research that more effectively promotes the uptake of liver cancer screening.* – Faculty Researcher

CPCRN = cancer prevention and control research network; TIDIRC = training institute for dissemination and implementation research in cancer; PPHEA = putting public health evidence into action training; FQHC = federally qualified health center; D&I = dissemination and implementation.

### Examples of Scholars’ research dissemination activities

Scholars were co-authors on 13 of 21 papers published in a CPCRN special supplement of *Cancer Causes & Control* published in 2023. The manuscripts focused on one of these five thematic areas: (1) Addressing Equity Through CPCRN, (2) Capacity Building, (3) Partnership Engagement, (4) Rural Cancer Prevention and Control, and (5) Future Cancer Needs and Priorities. Thirteen scholars were involved with presentations at the 16th Annual Conference on the Science of D&I co-hosted by the National Institutes of Health and Academy Health; nine scholars presented their projects at a national CPCRN network grantee meeting in May 2023.

## Discussion

The CPCRN Scholars program is grounded deeply in public health, community engagement, and health equity principles. The program has been highly effective in achieving enrollment goals and diversifying the D&I science expertise of public health researchers, practitioners, and students.

Senior mentors have been from a wide variety of academic backgrounds with skill sets and life experiences that were particularly attractive to scholars. For example, a scholar may have been in an epidemiology program; so, receiving guidance and new knowledge in CBPR methods from workgroup members and/or a mentor at another institution across the country was considered unique.

This is one of few programs that was established not only for advanced researchers but also for students, postdoctoral fellows, junior faculty members, and practitioners who may be new to D&I science. Related to this, the network has always been open to people’s project ideas – for workgroups, interest groups, and new projects. Applicants represented a wide range of characteristics, including scholars’ commitment to a particular project idea. As noted, some will use this as an opportunity to further develop their dissertation research related to cancer control or D&I; therefore, they would have a specific project idea. Others would have a general idea of where their interests might lie. These individuals often were “shopping” for training ideas and would often be directed to particular workgroups that would help them to realize these interests.

Scholars reported significant improvements in self-ratings of all competencies from baseline to post-program. These differences were greatest for items related to Practice-Based Considerations followed by Theory & Analysis. This was expected given the core competencies were linked to specific curricular content which emphasized Theory & Analysis and promoted engagement in individual D&I research projects that integrated hands-on experiential learning in CBPR and related practice-based competencies. The slightly lower improvements in basic D&I knowledge, as compared to these two areas, may reflect the higher baseline knowledge (overall median competency score 1.52) of theory, possibly a result of the program attracting scholars with some background awareness, knowledge, and vested interest in D&I knowledge and skill expansion.

### Lessons learned with regard to recruitment, process and implementation, and continued engagement and sustainability

#### Recruitment

As an academic research network, CPCRN was successful with recruitment of graduate students and postdoctoral fellows, for whom there are few mentored D&I programs at this training stage, but recruitment of investigators new to D&I research and cancer prevention and control practitioners from clinical and public health settings was more challenging. After the NCI’s TIDIRC facilitated program transitioned to non-mentored open access only in 2022 ^2^, the number of researchers participating in the Scholars Program increased. This provides evidence that the Scholars Program is filling a gap in desired mentored training for academic investigators.

For the second cohort, concerted outreach to networks of clinicians and public health professionals helped bolster the involvement of these key partners in the Scholars Program. D&I training for practitioners is critical to enhance their capacity to identify, adapt, implement, evaluate, and sustain evidence-based interventions in practice at a scale that will achieve positive population health outcomes. Further, the inclusion of practitioners from clinical and public health settings in D&I training increases their readiness to engage with D&I researchers in planning, conducting, and disseminating more relevant science. As argued by Smith and Wilkins [34], building a community of D&I-trained practitioner scholars who can lead practice-based research or collaborate in research-practice partnerships can help bridge the intractable gap between research and translation to practice. The inclusion of practitioners in this training helps meet a documented global gap in D&I training for these critical partners [12,35]. It remains challenging to recruit practitioners, however, we have made it a priority to collaborate with practitioners in program design and improvements to ensure we are meeting the needs of those in the field.

#### Process and implementation

The CPCRN Scholars Program, which was developed under the national CPCRN, is unique in identifying clinicians, practitioners, and researchers for training in D&I research. Recruitment was initially conducted across the United States and by the third cohort had expanded to include international applicants. Geographical dispersal made it essential to consider a remote and flexible approach to training to ensure the greatest opportunities for engagement in terms of time commitments and geographical reach. The program was designed to be self-paced to afford greater flexibility to allow working adults to participate and provide transparent expectations for deliverables and deadlines for task completion. The Trello board was selected as a project management tool wherein each scholar was made aware of tasks, dates, and deadlines. This ensured that key training communications and content were made available to all fellows in a timely manner. Real-time tracking aligned with competency acquisition also allowed early identification of any scholar needing additional training support.

The self-paced program placed the responsibility for project progress with the scholar. Individuals who, though competent and highly motivated, were generally faced with competing demands on their time. This challenge of time for training and project completion has been noted across D&I capacity-building programs [12]. For such capacity-building programs, mentor oversight in terms of regular meetings to evaluate progress and even written progress reports can help ensure timely project completion and greater time equity across scholar projects. Interestingly, a significant percentage of program scholars continued with their project beyond the 12-month training period, and many engaged in additional projects to further expand their training and mentoring opportunities in D&I science.

Evaluation data from exit interviews (with Cohort 1) and surveys (pre/post and alumni) as well as ongoing program feedback from scholars at network annual meetings suggested that some scholars were not receiving enough general professional development opportunities in their current professional setting. Among the interests voiced by scholars are training in grant budgeting, scientific writing, and how to prepare and deliver scientific presentations, and interview job talks. While professional development was beyond the scope of this D&I training program, many scholars received this guidance informally from their mentors and/or the program leadership and they identified the value of mentoring in professional development as a positive aspect of the training experience. To address this need without diluting the current focus, the planning workgroup developed an all-cohort professional development year for which they are offering virtual seminars and networking events guided by input provided by all three scholar cohorts via a training assessment survey. Some of the topics included a writing retreat for progressing on their papers or grant proposals, how to conduct systematic reviews and writing a D&I grant.

Evaluating the program using surveys with scholars and alumni and interviews with scholars and mentors was extremely helpful for ensuring ongoing quality assurance, introspection, and iterative program adaptation to meet scholars’ needs. Continuing to ensure data are collected from scholars and mentors and from planning workgroups moving forward will be helpful given that national workgroups engaged a number of scholars during this grant cycle and workgroup leads and investigators may have also served as informal mentors for scholars. We also recommend that training programs are creatively combined with data collection activities. Mixed methods evaluation with qualitative reflection and stories are very important for improvement and for the recruitment of future cohorts. Collecting such narratives in a meaningful way will help with the expansion and sustainability of the program. Of note, the program evaluation to date has largely focused on short-term impact as outlined in our logic model. Going forward the program will need to demonstrate greater intermediate and long-term outputs and impact to support its sustainability. In fact, a more comprehensive logic model-driven, outcomes-centric approach to evaluating self-competencies has been developed and shown to be effective in the training of clinical translational scientists [36–39].

Building on the CPCRN was a critical asset for the Scholar’s program. Beyond the curriculum and webinars, scholars were able to attend the annual CPCRN virtual and in-person meetings as its own infrastructure. This allowed them to network, contribute to the efforts of workgroups, share their own science, and build their network of D&I researchers and practitioners in cancer control. Learning more about the CPCRN and continued participation in the network was a common evaluation finding [25].

### Limitations with program implementation evaluation

There are important limitations to note with this research training program. First, we approached diversity broadly in how we defined and assessed it in the application rubric. The mentor/scholar match was done primarily based on content and topic expertise and the diversity of the research background and interests of the applicants. More intentional consideration of diversity for future scholar/mentor matching should be conducted as has been done in other research training programs [40,41]. Second, we did not examine all aspects of the program in our evaluations, despite the use of multiple assessments. For example, we only asked about CPCRN-relevant dissemination on the alumni survey. We also did not inquire about scholars’ satisfaction with specific curriculum content or evaluate knowledge and skills obtained beyond the self-related competency assessment on the pre- and post-program survey. Third, while the Trello board was a convenient platform for housing the program curriculum, in retrospect, leveraging this system to track training competency at the individual scholar level by aligning learning activities with knowledge acquisition and applications would have ensured all competencies were met over time by each scholar.

Fourth, while the self-rated competencies assessment is published and has been used in several D&I trainings, there are no validation studies. In addition, we recognize that other methods such as logic model competency identification or competency identification through competency-based educational frameworks can be used to establish cutoff points for competencies given the potential limitation of implementing a tertile ranking for this purpose [37,38]. Importantly, we did use overall domain scores to drive our interpretation of the evaluation results so as not to overinterpret our findings related to individual subdomain responses. Fifth, we recognize that with the cohort size, we were unable to perform additional meaningful statistics (subanalyses) that would be possible with a larger sample. We also had not administered the alumni survey with the third cohort of scholars at the time this paper was written. Finally, we conducted in-depth interviews only with the initial cohort of scholars. While these interviews helped inform future years of the program, it is recommended that a more consistent collection of qualitative data be implemented with scholars, mentors, and workgroups.

## Conclusions

This paper has described evaluation data and lessons learned about a formal training program of a CDC-funded research network. While the focus of this program has been on cancer prevention and control and D&I science, the process of program development, implementation, and evaluation as well as recommendations can be applied to other mentored capacity-building initiatives that collaborate across multiple institutions. Sustaining such a training program is important and deserves the thoughtful attention of planners [42]. The scale and scope of such a training program may deserve its own dedicated funding support to allow for further touch points (in-person meetings) or programming. The infrastructure of having a collaborative network of centers with a strong coordinating center is critical for the continued success of the CPCRN Scholars Program.

We plan to keep up the momentum and enthusiasm for this program through continued engagement with scholar alumni and mentors. We will do this by asking scholars to help recruit future cohorts, serve in mentor roles after completing the program, and remain engaged through participation in panels, webinars, and conference presentations. Encouraging continued collaboration on projects through national workgroups will also keep scholars engaged with prior and new mentors and collaborators and allow them to be part of initiatives that benefit their career trajectories regarding academic deliverables and access to community and other partner groups. Ongoing scholar assessment and evaluation will allow for building in preferred training topics and components that will be valuable to their career trajectory and make a public health impact. Future training initiatives are still needed to build capacity in D&I competencies and science and continue to improve public health in communities.
